# Source of the Phosphates in Bone

**Published:** 1865-12

**Authors:** 


### SOURCE OF THE PHOSPHATES IN BONE.

---

Large masses of phosphorus are, says Dr. Hoffman's Report, in the course of geological revolutions, extending over vast periods of time, restored from the organic regions of nature to the mineral kingdom by the slow process of fossilization; whereby vegetable tissues are gradually transformed into peat, lignite, and coal; and animal tissues are petrified into coprolites which in course of time yield crystalline apatite. After lying locked up and motionless in these forms for indefinite periods, phosphorus, by further geological movements, becomes again exposed to the action of its natural solvents, water and carbonic acid, and is thus restored to active service in the organisms of plants and lower animals, through which it passes, to complete the mighty cyle of its movements into the blood and tissues of the human frame. While circulating thus, age after age, through the three kingdoms of nature, phosphorus is never for a moment free. It is throughout retained in combination with oxygen, and with the earthy or alkaline metals for which its attraction is intense.—*Report on Chemical Products in Exhibition of* 1862.

